# Soil pH: a key edaphic factor regulating distribution and functions of bacterial community along vertical soil profiles in red soil of pomelo orchard

**DOI:** 10.1186/s12866-022-02452-x

**Published:** 2022-02-02

**Authors:** Muhammad Atif Muneer, Wei Hou, Jian Li, Xiaoman Huang, Masood ur Rehman Kayani, Yuanyang Cai, Wenhao Yang, Liangquan Wu, Baoming Ji, Chaoyuan Zheng

**Affiliations:** 1 College of Resources and Environment, International Magnesium Institute, Fujian Agriculture and Forestry University, Fuzhou, 350002 China; 2 Center for Microbiota and Immunological Diseases, School of Medicine, Shanghai General Hospital, Shanghai Institute of Immunology, Shanghai Jiao Tong University, Shanghai, 200025 China; 3 College of Grassland Science, Beijing Forestry University, Beijing, 100083 China

**Keywords:** Red soil, Bacterial diversity, Functional analysis, Soil pH, Spatial distribution

## Abstract

**Background:**

Soil microbes exist throughout the soil profile and those inhabiting topsoil (0–20 cm) are believed to play a key role in nutrients cycling. However, the majority of the soil microbiology studies have exclusively focused on the distribution of soil microbial communities in the topsoil, and it remains poorly understood through the subsurface soil profile (i.e., 20–40 and 40–60 cm). Here, we examined how the bacterial community composition and functional diversity changes under intensive fertilization across vertical soil profiles [(0–20 cm (RS1), 20–40 cm (RS2), and 40–60 cm (RS3)] in the red soil of pomelo orchard, Pinghe County, Fujian, China.

**Results:**

Bacterial community composition was determined by 16S rRNA gene sequencing and interlinked with edaphic factors, including soil pH, available phosphorous (AP), available nitrogen (AN), and available potassium (AK) to investigate the key edaphic factors that shape the soil bacterial community along with different soil profiles. The most dominant bacterial taxa were *Proteobacteria*, *Acidobacteria*, *Actinobacteria*, *Chloroflexi*, *Crenarchaeota,* and *Bacteriodetes*. Bacterial richness and diversity was highest in RS1 and declined with increasing soil depth. The distinct distribution patterns of the bacterial community were found across the different soil profiles. Besides, soil pH exhibited a strong influence (pH ˃AP ˃AN) on the bacterial communities under all soil depths. The relative abundance of *Proteobacteria, Actinobacteria, Crenarchaeota,* and *Firmicutes* was negatively correlated with soil pH, while *Acidobacteria, Chloroflexi, Bacteriodetes, Planctomycetes,* and *Gemmatimonadetes* were positively correlated with soil pH. Co-occurrence network analysis revealed that network topological features were weakened with increasing soil depth, indicating a more stable bacterial community in the RS1. Bacterial functions were estimated using FAPROTAX and the relative abundance of functional bacterial community related to metabolic processes, including C-cycle, N-cycle, and energy production was significantly higher in RS1 compared to RS2 and RS3, and soil pH had a significant effect on these functional microbes.

**Conclusions:**

This study provided the valuable findings regarding the structure and functions of bacterial communities in red soil of pomelo orchards, and highlighted the importance of soil depth and pH in shaping the soil bacterial population, their spatial distribution and ecological functioning. These results suggest the alleviation of soil acidification by adopting integrated management practices to preserve the soil microbial communities for better ecological functioning.

**Supplementary Information:**

The online version contains supplementary material available at 10.1186/s12866-022-02452-x.

## Background

Soil is one of the most diverse ecosystems consisting of microorganisms, organic matter, minerals, and water [1]. Soil microbes, including bacteria, fungi, macrofauna, mesofauna, and microfauna (e.g., protozoans and nematodes), are considered as the major soil biota [2]. Among these, bacteria have the most abundant community structure in the soil [3]. The soil microbial diversity is a distinguishing attribute of the agricultural systems and a variety of biogeochemical cycles, e.g., organic matter decomposition and soil nutrients cycling [4–6]. Therefore, it is necessary to understand the community composition and its response to environmental changes that could significantly affect the ecological functions.

Despite the widespread occurrence of soil microbiota in different soil depths, the current knowledge about the distribution of soil microbes is limited to surface soil (0—20 cm) [7] owing to higher contents of soil organic matter, mineral nutrients, and rich in microbial diversity compared with subsurface soil [8]. Various studies have investigated the microbial diversity and community composition in the surface and subsurface soils, e.g., in paddy soils or Alaskan soil cores [9–11]. The subsurface soil microbial community has great significance owing to its role in the soil formation process relative to the surface soil profile [12]. The soil microbial communities in subsurface soil also play a crucial role in soil carbon sequestration due to the storage of organic carbon in the subsurface soil profiles [13]. Thus, characterization of the soil microbial communities along various soil gradients would enable us to better understand the key characteristic of soil microbial communities and their potential functions in the red soil.

Red soil is mainly distributed in the southern regions of China (2.18 × 10^8^ ha), covering 22% of the total land area [14]. These soils are characterized by low pH, low nutrient contents, and high aluminum ion activity that results in poor soil physical properties that are key limiting factors for plant growth [15], especially for pomelo orchards in the red soil of Pinghe County (Fujian Province), which is the most famous area for pomelo production [16, 17]. The cultivated area and yield of pomelo have been increased in recent decades, and total production of pomelo reached over 5 × 10^4^ ha with an annual production of over 130 × 10^4^ t (Pinghe County Statistical Yearbook, 2017). Recently, excessive use of fertilizers for higher yield has resulted in serious environmental problems such as soil acidity [18], soil contamination [19], greenhouse gases emission [20], water contamination [21], and detrimentally influenced the soil microbial diversity [22]. Evidence suggests that the soil microbial community has a significant role in the maintenance of soil structure and nutrients cycling, hence soil microbial diversity is an important index to assess soil health [23]. The diversity and community composition of soil microbes are significantly influenced by soil type, fertilizer type, application methods, and other various factors [23, 24]. For example, the application of inorganic fertilizers, especially N-fertilizer plays a significant role in improving crop productivity, however, its long-term application deteriorates crop productivity and soil quality [25, 26]. Nevertheless, the contradictions and uncertainness persist regarding the substantial impacts of inorganic fertilizers on the functional diversity and composition of soil microbes. Besides, inorganic fertilizers have a significant effect in improving biomass carbon and nitrogen [27], but decreases the functional diversity of soil microbes under long-term N application [28]. Some studies have found no effect of N application on community composition and functional diversity of soil microbes [24, 29]. In contrast, the inorganic fertilizers have shown the direct effects on functional diversity as well as community composition of soil microbiota [30]. However, in pomelo orchards, the effects of intense inorganic fertilization on soil microbial richness and community structure have not been fully elucidated.

Although several previous findings have validated the potential role of soil microbes in soil functioning and ecosystem services under various ecological conditions [31–33], however, very limited information is available about the important edaphic factors of soil bacterial community structure and functional diversity in the red soils. Hence, we investigated the bacterial diversity and community structure by 16S rRNA sequencing and conducted detailed analyses for microbial community structure through co-occurrence networks and prediction of functional studies. The key research objectives were as follows: (1) to explore the distribution of bacterial diversity and community composition along with the vertical soil profile under intensive use of fertilizers in red soil; (2) to investigate the important edaphic factors that shape the soil bacterial community; (3) to predict the functional potential of bacterial community and their relationship with the edaphic factors.

## Results

### Changes in soil physicochemical properties under different soil depths

**Fig. 1:**
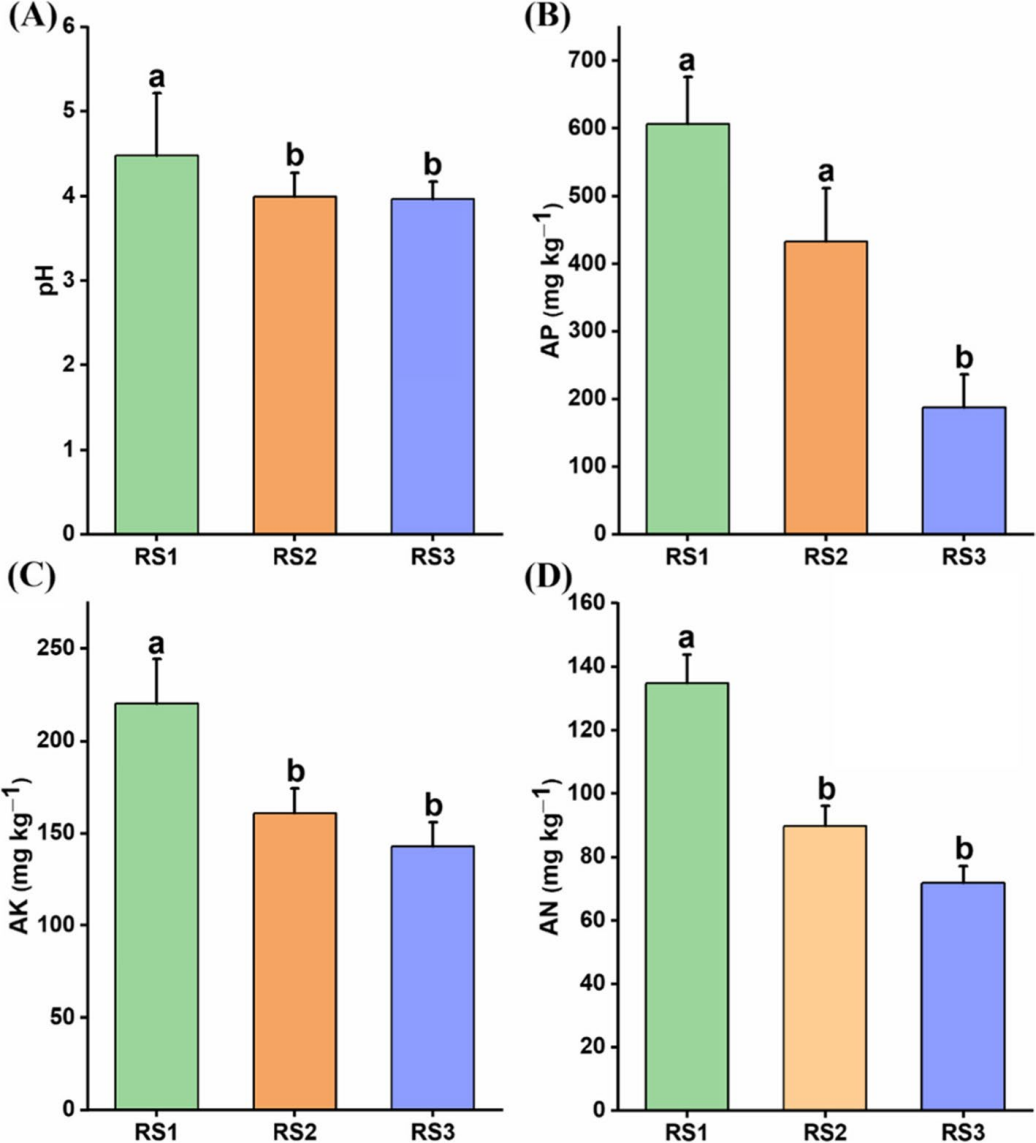
Changes in soil physicochemical properties. The variation in soil physicochemical properties were observed for; **A** Soil pH; **B** Available nitrogen (AN); **C** Available phosphorous (AP); **D** Available potassium (AK) along vertical soil depths. The different lowercase letters indicate the significant differences (*p* < 0.05) among different soil depths, i.e., RS1 (0–20 cm), RS2 (20–40 cm), RS3 (40–60 cm)

Soil physicochemical properties were significantly affected along soil profile gradient. Soil pH was significantly higher in topsoil RS1 (4.47) compared with RS2 (3.99) and RS3 (3.96) (Fig. 1A). Similarly, the available nutrients contents including AN (Fig. 1B), AP (Fig. 1C), and AK (Fig. 1D) were also significantly higher in RS1 compared with RS2 and RS3. Overall, we found that with decreasing the soil pH, the availability of the nutrients also decreased.

### Effect of soil depth on specific bacterial community

**Fig. 2:**
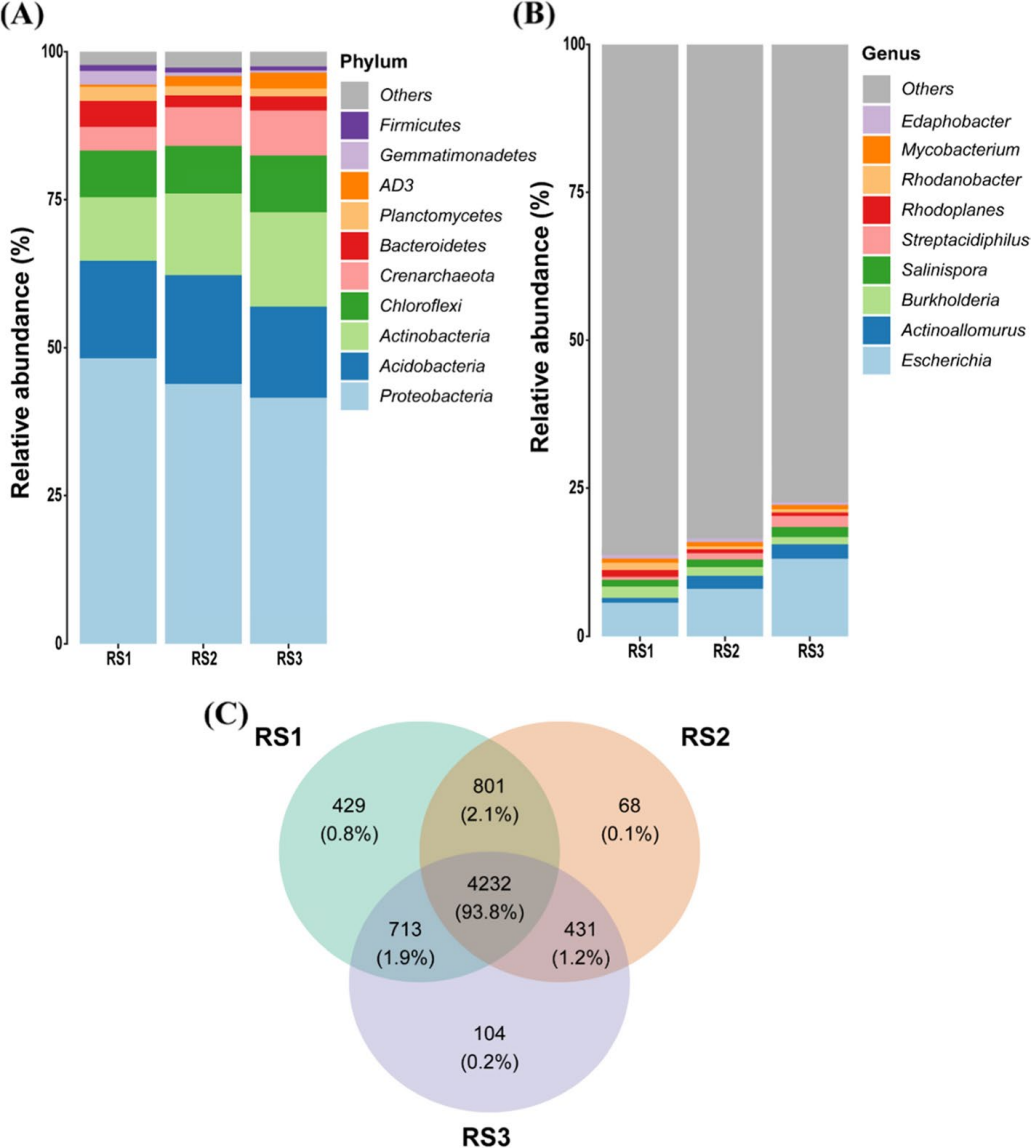
Relative abundance of bacterial communities in three soil depths. The relative abundance of dominant bacterial communities; **A** Phylum level; **B** Genus level; **C** Comparison of bacterial communities’ richness in different soil depths, i.e., RS1 (0–20 cm), RS2 (20–40 cm), RS3 (40–60 cm). The relative abundance of top 10 phylum/Genus has been shown, while the unclassified and less abundant were classified as others

In total, 6778 OTUs were extracted from 57 soil samples and categorized based on phylum (34), class (109), order (214), family (345), and genus (598). The dominant phyla included the *Proteobacteria*, *Acidobacteria*, *Actinobacteria*, *Chloroflexi*, *Crenarchaeota*, and *Bacteriodetes*, which accounted for 92% of all microbial communities (Fig. 2A). The relative abundance (RA) of *Proteobacteria*, *Acidobacteria,* and *Bacteriodetes* decreased with increasing soil depth, while RA of *Actinobacteria* and *Crenarchaeota* increased along with the soil depth, maximum at 40–60 cm (RS3) depth soil layer (Supplementary Fig. S1). Similarly, the differences in bacterial communities were observed at the genus level (Fig. 2B). Overall, these findings show that changes at the phylum level for specific bacterial are contributed by the soil depth. According to the Venn diagram, 4232 OTUs were found to be prevalent across the three soil depths (Fig. 2C) and accounted for 93.8% of the total number of reads. It implies that the similarity of bacterial community composition was high in all depths of the red soil.

### Richness and diversity of bacterial communities decreased along the soil profile gradient

**Fig. 3:**
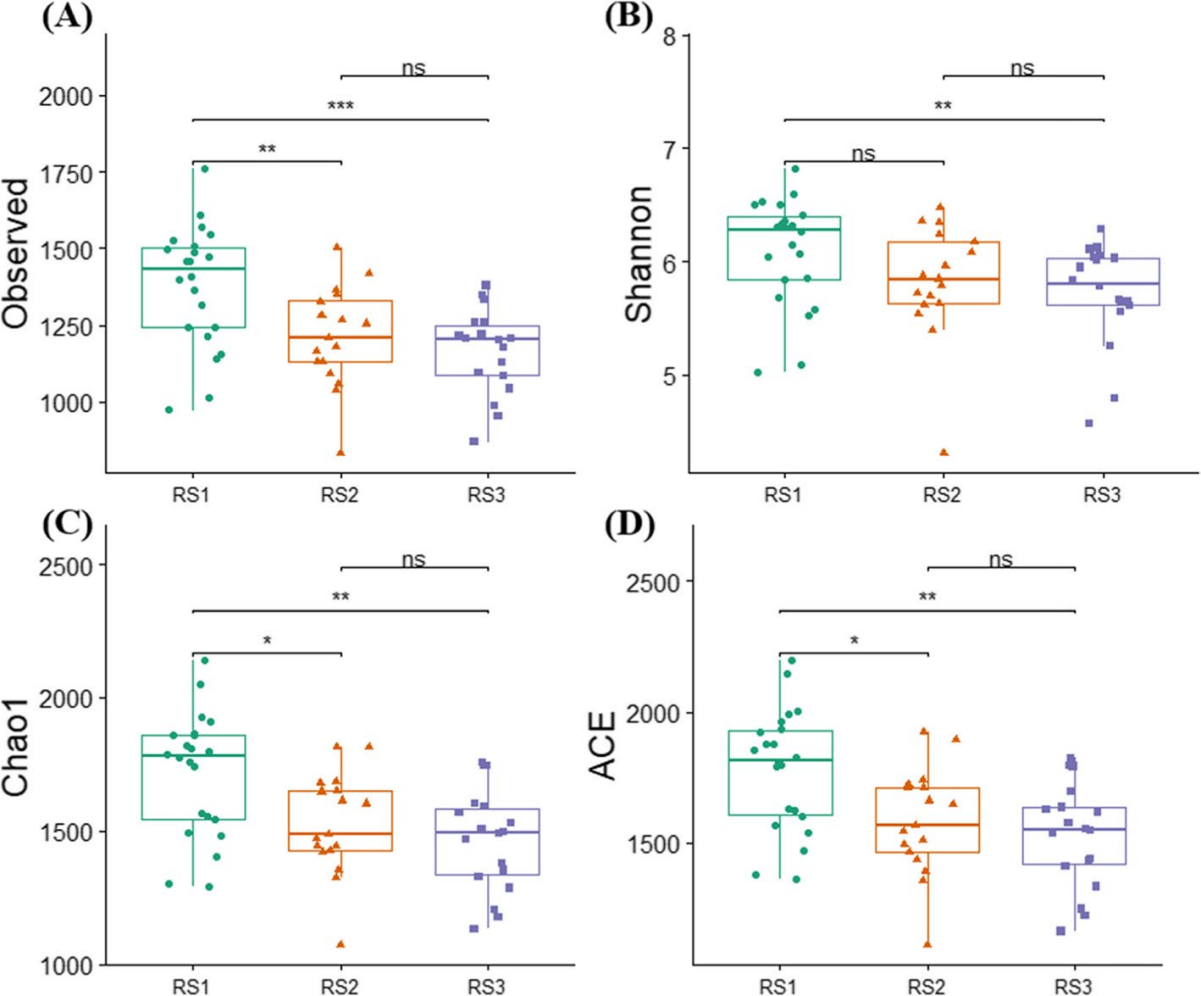
The α-diversity indices in different soil depths. The α-diversity indices were calculated; **A** Observed OTUs; **B** Shannon index; **C** Chao1; **D** ACE index. The differences between any two soil depths were tested by Wilcon test. The star represents the significance (*, *P* ≤ 0.05; **, *P* ≤ 0.01; ***, *P* ≤ 0.001, while ns, non-significant. RS1, RS3, and RS3 represents the soil depths of 0–20, 20–40, and 40–60 cm, respectively

We assessed the four α-diversity indices, including the observed number of OTUs, Shannon index, Chao1 index, and ACE index. The species richness indices, i.e., the observed number of OTUs, Chao1 and ACE were significantly higher in the RS1 (0–20 cm) than RS2 (20–40 cm), and RS3 (40–60 cm) soil depth. Similarly, Shannon's was consistent and found the higher diversity in RS1, and it was significantly different from the RS3 (Fig. 3). It implies that bacterial richness and diversity were high in the topsoil and declined with increasing soil depth.

### Changes in bacterial communities across the soil depth

**Fig. 4:**
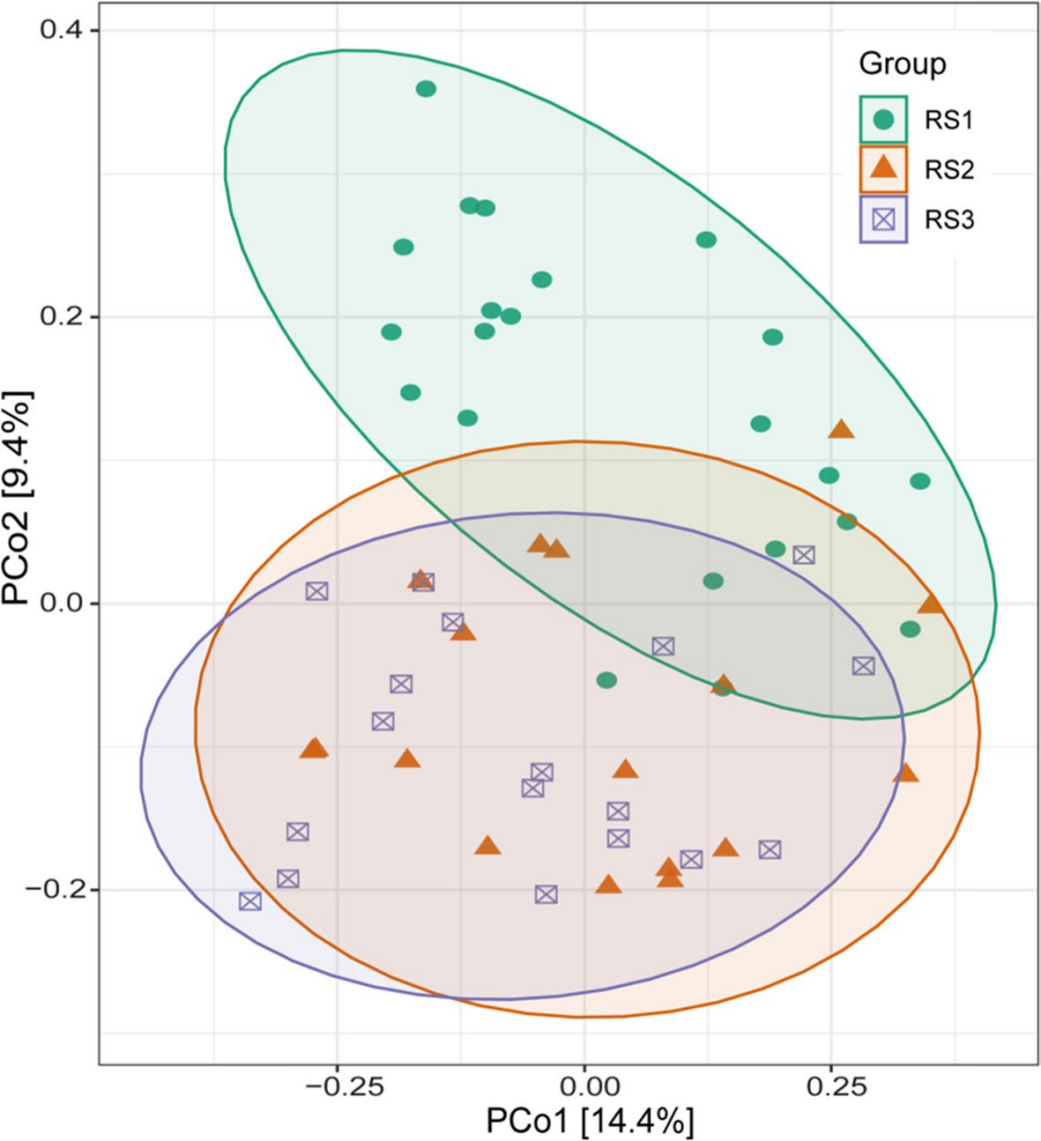
Changes in bacterial community structures and composition. Principal coordinate analysis (PCoA) analysis showing the differences in bacterial communities among different soil depths. RS1, RS3, and RS3 represents the soil depths of 0–20, 20–40, and 40–60 cm, respectively

To assess whether the soil depth influenced the microbial community composition across the different soil depths, the principal coordinates analysis (PCoA) was performed. The results showed that bacterial communities were separated by the different soil depths, where PCo1 accounted for 14.4% and PCo2 9.4% of the variation (Fig. 4). PERMANOVA analysis of the complete dataset suggested the significant differences in bacterial communities of different soil depths (Table 1). Furthermore, we used PERMANOVA analysis to analyze the differences between the different soil depths for each paired group and discovered that bacterial populations were segregated by soil depth (Table 2). Soil bacterial communities in the RS1 were significantly different than RS2 and RS3, while no significant difference was found between the RS2 and RS3 bacterial communities (Table 2). These findings showed that soil depth has a significant effect on bacterial community composition.

**Table 1:** Permutational analysis of variance (PERMANOVA) for all groups

	**Df**	**Sum of sqs**	**Mean Sqs**	**F. Model**	**R**^**2**^	**Pr(> F)**
Group	2	1.249	0.624	2.717	0.092	**0.001**
Residuals	54	12.418	0.229		0.908	
Total	56	13.668			1	

Significant *P*-value (≤ 0.05) is bolded

**Table 2:** Significance test of differences among bacterial communities in different soil depths using permutational analysis of variance based on Bray–Curtis distance

Groups	Measure	Permutations	R^2^	P. value	Significance
RS1 vs RS2	bray	999	0.064	0.002	***
RS1 vs RS3	bray	999	0.0956	0.001	**
RS2 vs RS3	bray	999	0.0428	0.066	

The star represents the significance (**, *P* ≤ 0.01; ***, *P* ≤ 0.001)

### Soil properties correlated with bacterial community

**Fig. 5:**
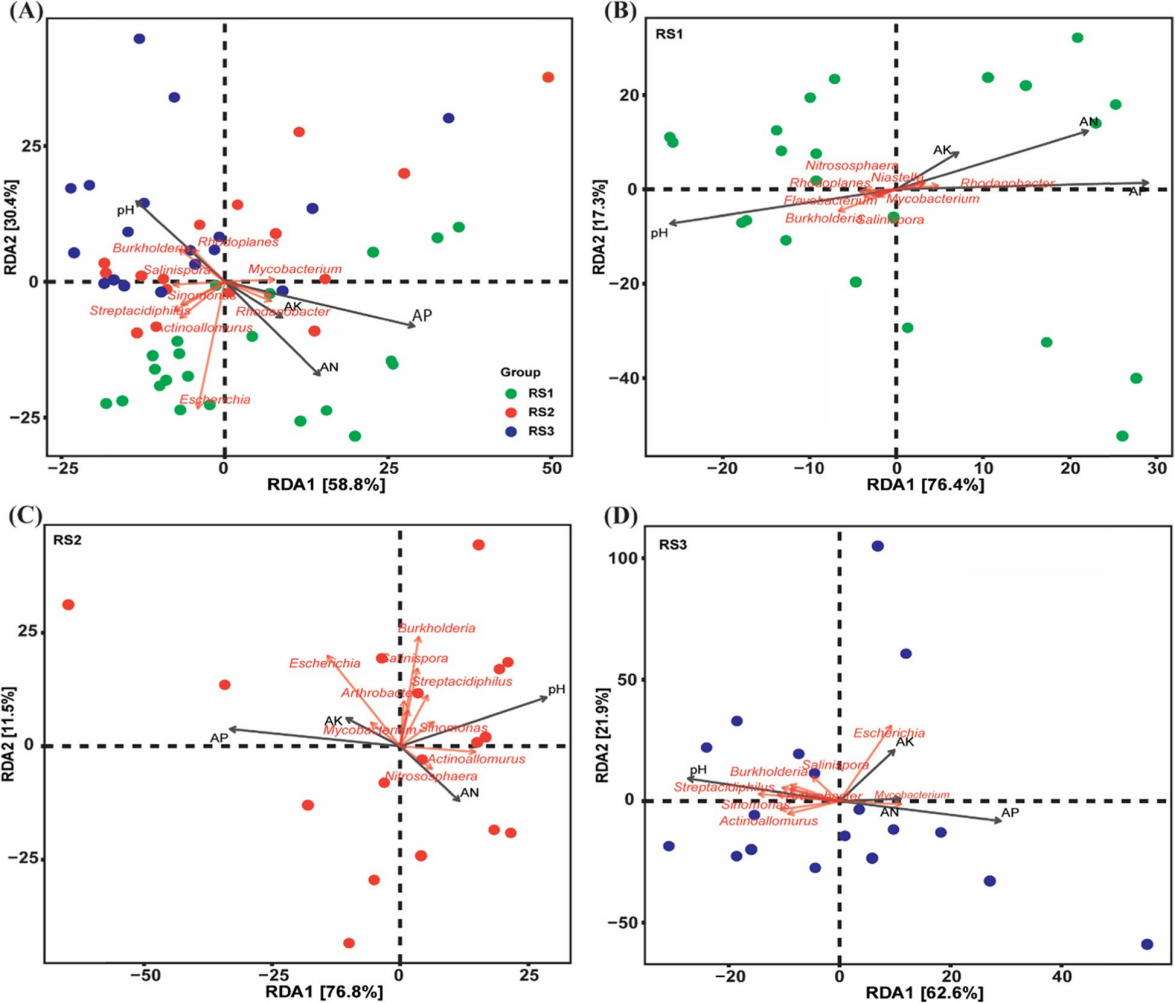
Effect of soil properties on bacterial communities. The distance-based redundancy analysis (RDA) ordination plot of bacterial communities based on community-environment relationship; **A** overall for all soil depths; **B** soil depth RS1 (0–20 cm); **C** soil depth RS2 (20–40 cm); **D** soil depth 40–60 cm

**Fig. 6:**
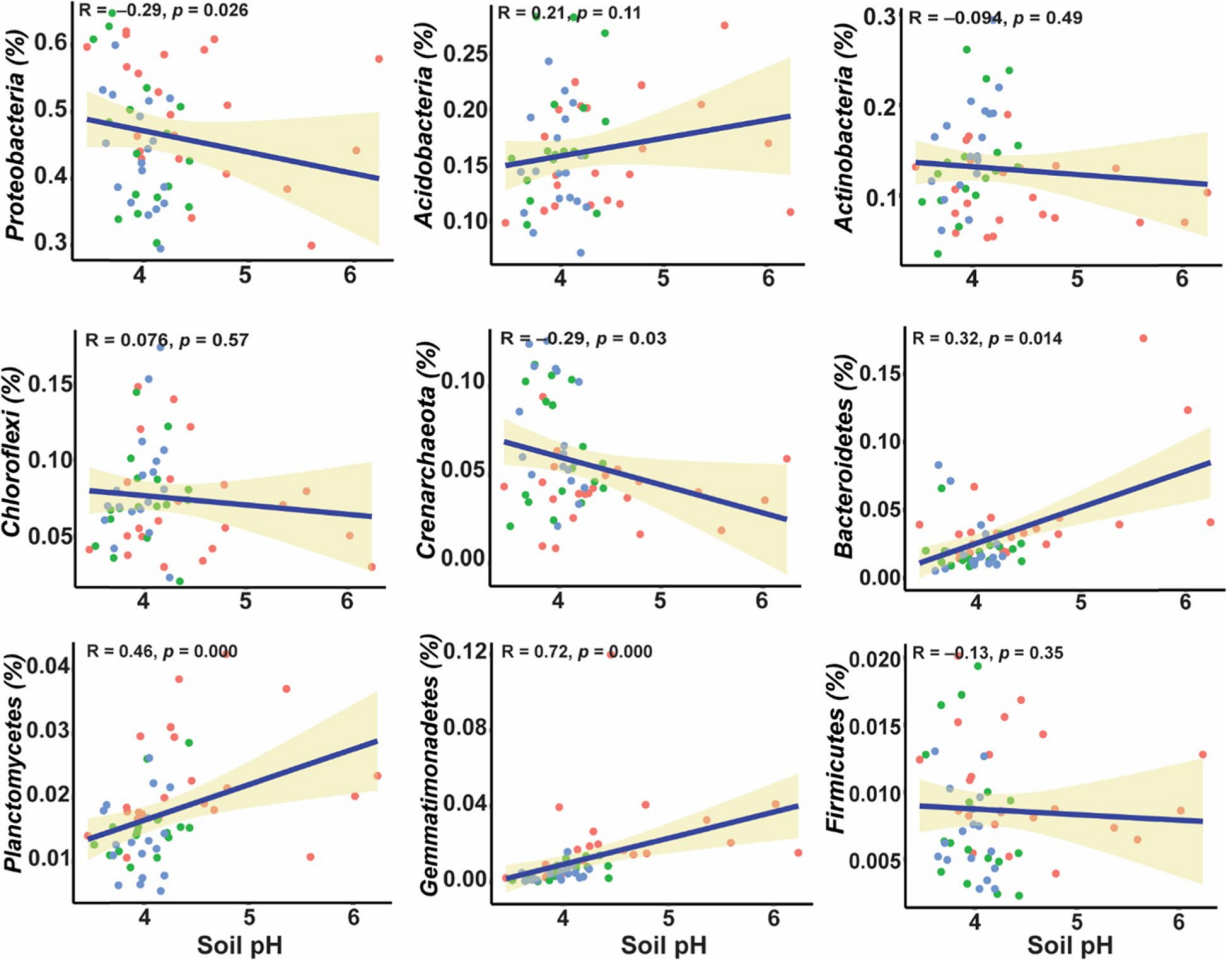
Correlation analysis between the soil pH and bacterial communities. The effect of soil pH was tested on abundant taxa of bacterial community at phylum level

The distance-based redundancy analysis (RDA) exhibited that all the soil properties except AK, were significantly positively correlated in shaping the bacterial community’s structure, and the soil pH, AP, and AN had significant effects in changing the bacterial community’s structure (Fig. 5A). Among these edaphic factors, we found that bacterial populations were most affected by soil pH in the red soils (Table 3). Furthermore, we investigated these effects in each soil depth and found that soil pH was the most influential factor affecting the soil bacterial communities in each soil depth, including RS1 (Fig. 5B), RS2 (Fig. 5C), and RS3 (Fig. 5D), Table 3. Correlation analysis was further performed to explore the effect of soil pH on each bacterial community. Overall, we found that the relative abundance of *Proteobacteria*, *Actinobacteria*, *Crenarchaeota*, and *Firmicutes* was negatively affected by soil pH. In contrast, soil pH had a positive effect on the relative abundance of *Acidobacteria*, *Chloroflexi*, *Bacteriodetes*, *Planctomycetes*, and *Gemmatimonadetes*. Hence, soil pH plays a significant role in shaping the soil bacterial communities (Fig. 6).

**Table 3:** Pearson correlation between the Bray–Curtis dissimilarity score and soil characteristics using the mantel test

	**Variable name**	**Corr-method**	**Corr_res**	**p_res**	**significance**
**Overall**	pH	Pearson	0.366	0.001	***
	AP (Available Phosphorous)	Pearson	0.217	0.002	**
	AK (Available Potassium)	Pearson	-0.007	0.466	
	AN (Available Nitrogen)	Pearson	0.127	0.037	*
**RS1**	pH	Pearson	0.403	0.008	**
	AP (Available Phosphorous)	Pearson	0.113	0.132	
	AK (Available Potassium)	Pearson	-0.182	0.993	
	AN (Available Nitrogen)	Pearson	-0.047	0.639	
**RS2**	pH	Pearson	0.288	0.008	**
	AP (Available Phosphorous)	Pearson	0.322	0.005	**
	AK (Available Potassium)	Pearson	-0.226	0.963	
	AN (Available Nitrogen)	Pearson	0.224	0.087	
**RS3**	pH	Pearson	0.192	0.008	**
	AP (Available Phosphorous)	Pearson	0.247	0.005	**
	AK (Available Potassium)	Pearson	-0.163	0.963	
	AN (Available Nitrogen)	Pearson	-0.119	0.087	

The star represents the represented the significance (*, *P* ≤ 0.05; **, *P* ≤ 0.01; ***, *P* ≤ 0.001)

### Co-occurrence network analysis

**Fig. 7:**
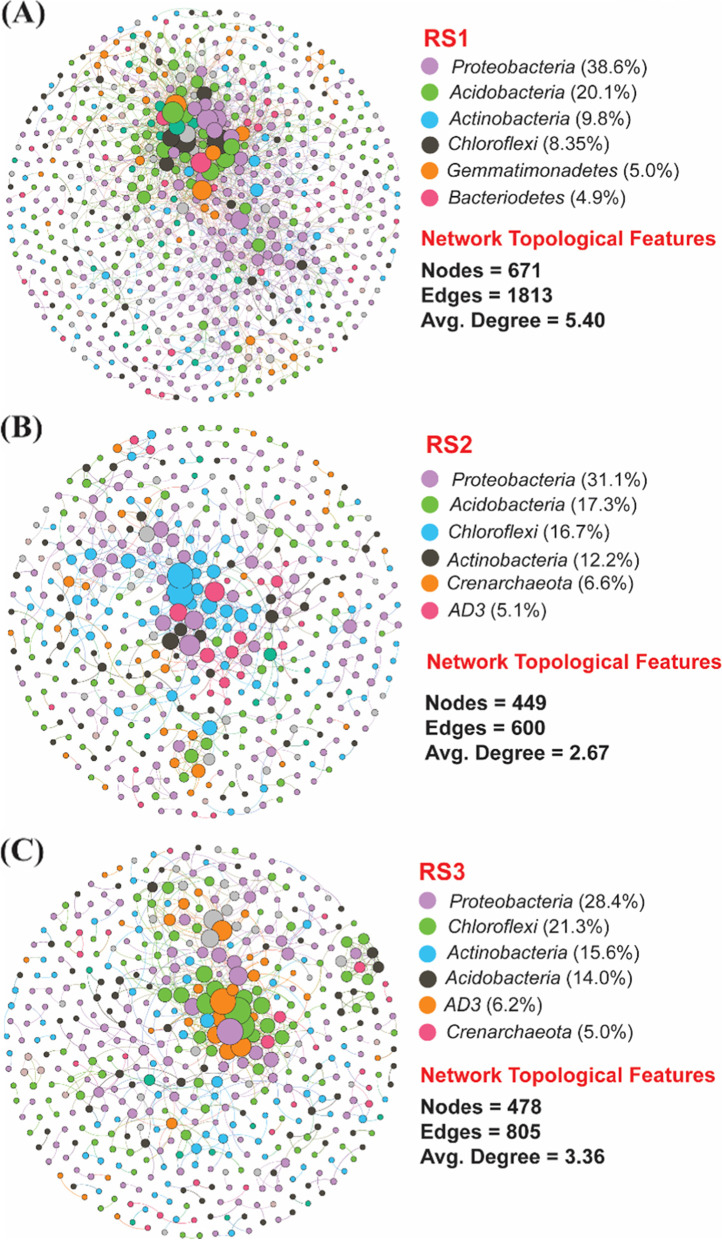
Co-occurrence network of bacterial communities in red soil based on correlation analysis. The Co-occurrence network of bacterial communities was investigated in **A** 0–20 cm (RS1); **B** 20–40 cm (RS2); **C** 40–60 cm (RS3). The nodes in the network are colored based on phylum. Size of each node is proportional to the relative abundance of specific taxa. The connections showing the strong (spearman's ρ ≥ 0.6) and significant (*P* ≤ 0.05) correlations

The taxonomic properties of the bacterial populations in the red soil were studied using a co-occurrence network analysis. In all soil depths, the network analysis revealed a significant correlation between the bacterial communities. The high-abundance nodes in the co-occurrence network were separated into six phyla. Among them, *Proteobacteria*, *Acidobacteria*, *Actinobacteria*, *Chloroflexi*, *Gemmatimonadetes,* and *Bacteriodetes* represented the most dominant bacterial community at phylum level in RS1. Meanwhile, topological properties were calculated to explore the complex interrelationship patterns among the nodes [34]. RS1 had the highest number of nodes and edges (671 and1813, respectively, Fig. 7A) compared with RS2 (449 and 600, respectively, Fig. 7B) and RS3 (478 and 805, respectively, Fig. 7C). It revealed that the soil bacterial network was more complex and bacterial associations were tight in RS1, while in RS2 and RS3 bacterial networks were less complex and bacterial associations were less tight.

### Functional analysis

**Fig. 8:**
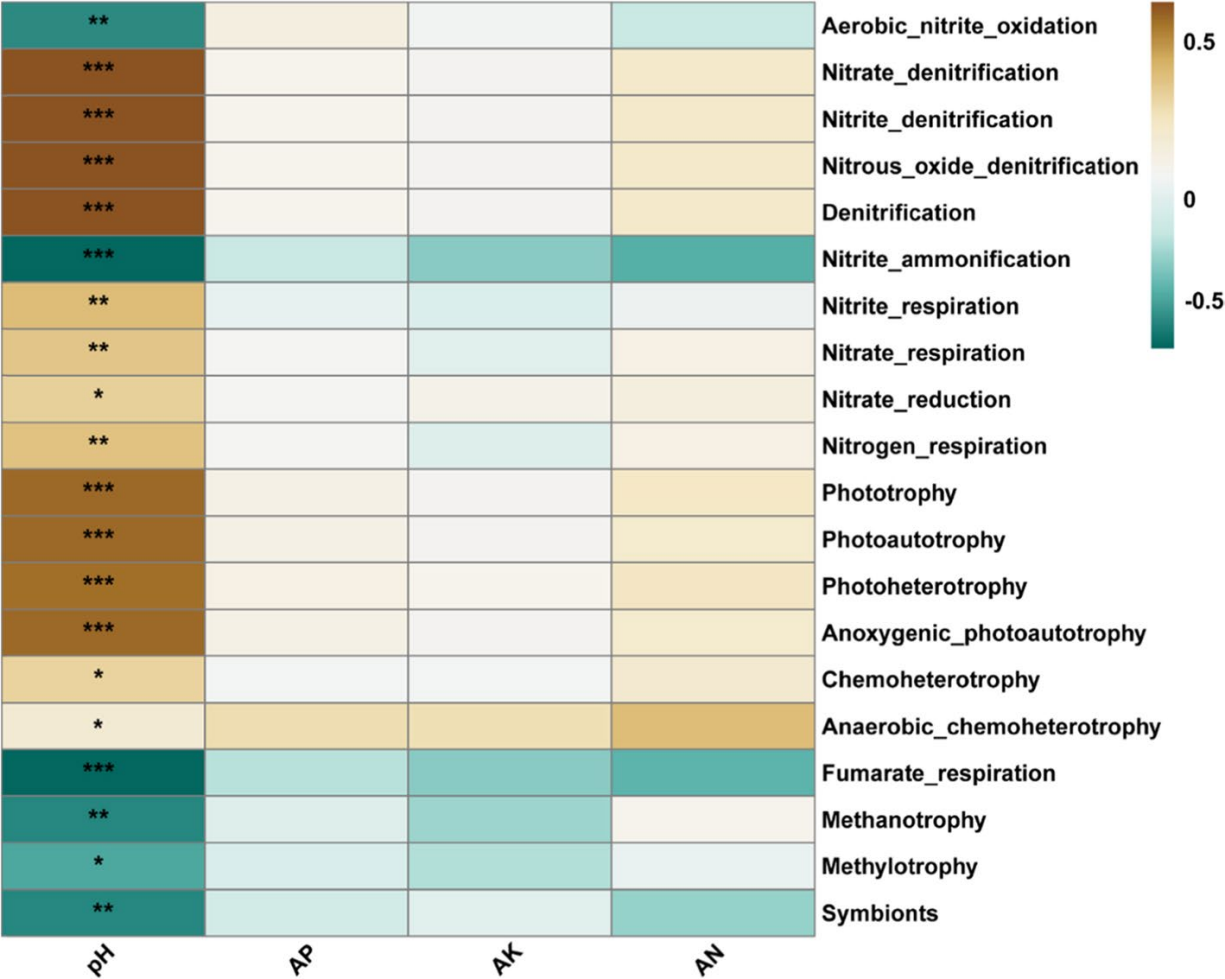
Effect of soil variables on functional microbial community. The potential role of edaphic factors was explored on metabolic processes regulated by bacterial groups. Heat map exhibiting the relative abundance of dominant function groups, and strength of correlation with the soil properties, including soil pH, available phosphorous (AP), available potassium (AK), and available nitrogen (AN). The star represents the significance (*, *P* ≤ 0.05; **, *P* ≤ 0.01; ***, *P* ≤ 0.001. The scale bar representing the strength of correlation

The expected functions of soil bacterial communities in pomelo orchards were determined using FAPROTAX analysis and predicted major functions were majorly attributed by C-cycle, N-cycle, and energy and their relative abundance were higher under RS1 compared with RS2 and RS3 (Supplementary Fig. S2). When the minor functions were predicted with the soil factors, we found that the metabolic functions of the C-cycle (e.g., photoautotrophy, photohetrotrophy, etc.), N-cycle (denitrification, nitrate and nitrite denitrification, etc.) were significantly affected by the soil pH (Fig. 8).

## Discussion

The bacterial community is most dominated in the soil habitat owing to their diversity and functioning in different soil biochemical processes [35]. Their community composition, spatial patterns, and functional profiling have yet to be well investigated in the red soil of pomelo orchards. Recent researches of soil microbial biodiversity and long-term fertilizer studies have revealed that soil bacterial diversity is significantly affected by the soil depth and edaphic factors that shape the soil bacterial community [7]. For example, soil pH is a primary determinant of microbial community composition and functional diversity [31, 36]. Thus, in the current study, we examined the bacterial community composition and functional diversity along with different soil profiles and identified the key edaphic factors regulating the soil bacterial diversity.

We found that different soil profiles representing variation in edaphic factors, e.g., soil pH, AN, AP, and AK were significantly higher in RS1 compared with RS2 and RS3 (Fig. 1), and had significant effects on bacterial community structure (Fig. 2). These findings are in concordance with the previous studies that soil depth plays an important role to shape the soil bacterial communities owing to differences in soil properties [7, 37]. Although soil properties (e.g., pH, AP, AK, AN) along different soil depths varied from each other but resulted in a high percentage of the shared OTUs accounted for 93.8% of total reads and it implies that similarity of bacterial community composition was high along the soil gradient. On the contrary, some taxa were still significantly different from each other in different soil profiles. For example, *Proteobacteria* and *Bacteriodetes* showed higher relative abundance in RS1 (Supplementary Fig. S1), and these are often categorized as copiotrophic group [38] and shows the higher growth rate with abundant resources [39], while *Acidobacteria* and *Chloroflexi* belonging to the oligotrophic group are highly abundant under low nutrients environment [40]. Hence, this is the reason that we found the higher relative abundance of *Proteobacteria* and *Bacteriodetes* in RS1 and *Acidobacteria* and *Chloroflexi* in RS3. So, these changes in bacterial communities along the soil depth could be attributed to differences in the soil properties [41]. The previous findings also showed the change in the relative abundance of bacterial communities along with the soil depth [42], although the changes in abundance patterns of bacterial communities were highly inconsistent. For instance, the relative abundance of *Acidobacteria* and *Actinobacteria* varied throughout the soil depth in one previous study [43]. However, we found that the RA of *Acidobacteria* was decreased with increasing soil depth, whereas *Actinobacteria* RA was at the peak in RS3 (40–60 cm), and consistent with the previous results where similar trends were observed in agricultural soils of Iowa, USA [7].

The differences in bacterial diversity and community composition were also revealed by α-diversity indices. The α-diversity of RS1 was significantly different from RS2 and RS3, but we found that RS2 and RS3 showed no difference in bacterial community structures although the differences in edaphic factors were high including pH, AP, and AK. However, the average soil pH was about the same in RS2 and RS3, i.e., 3.99 and 3.96, and as a result, we couldn't find differences in RS2 and RS3. Overall, we found that the species richness and alpha diversity was decreased along the soil depth gradient and consistent with the previous findings of Hao et al. (2020) who also found the decrease in bacterial diversity along with soil profile [7]. Furthermore, redundancy analysis revealed that soil pH had a substantial impact on the structure of bacterial communities (Fig. 5, Table 3), and the importance of soil pH in shaping the bacterial populations along with vertical soil profile has long been recognized [44]. A similar relationship has also been cited in previous studies [45, 46]. Schreiter et al. (2014) explained that besides some available nutrients, soil pH also drives the soil bacterial communities [45]. Sun et al. (2015) has demonstrated that soil pH is the key factor in shaping the soil bacterial communities [46]. The correlation analysis also showed that soil pH was a key factor for determining the soil microbial community composition, and our results agree with numerous previous studies [47, 48]. The close relationship of soil pH with the bacterial community is owing to the dependency of the majority of the bacterial community under narrow pH [49], and the slight change in pH significantly affects the bacterial community [50]. Many studies have reported the response of specific bacterial taxa to soil pH [51]. For instance, a negative correlation is found between the relative abundance of *Proteobacteria* and soil pH [52], a positive correlation between *Acidobacteria* and soil pH [53], we also found similar results in the present study. Furthermore, *Gemmatimonadetes* also showed a positive correlation with soil pH in the present study and supported by the results of Yang et al. (2020) [52].

Soil microorganisms, in general, do not function individually and instead establish a complex relationship network. Therefore, the relationship between diverse soil microbes has played a significant role in the functioning and stability of the microbial ecosystems [54]. The use of network analysis is a powerful technique for better understanding how related soil microbiomes adapt to environmental changes. In the current research, the network analysis revealed that bacterial taxa belonging to the phylum *Proteobacteria*, *Acidobacteria*, *Actinobacteria* were most dominant in the red soil, and consistent with the previous studies [55]. The highly connected nodes in RS1 compared with RS2 and RS3, revealed more complex and bacterial association in RS1, and such taxa are predictable as keystone bacterial taxa [54]. These keystones taxa are very crucial for maintaining the co-occurrence network structure [56]. Hence, the bacterial community in RS1 was more tolerant to environmental disturbances owing to complex connectivity because it has been widely accepted that bacterial communities with higher connectivity are more resilient to environmental changes compared with weaker connectivity of simple networks [57].

Numerous studies have confirmed that the functional composition of soil microbiota rather than taxonomic composition in natural environments appears to be closely related to environmental factors [58–60]. Although, the ecological functions of soil microbes inhabiting similar environments are closely related but their community composition may differ greatly. Hence, it implies that besides identifying which soil microorganisms are present in the environment, it is also a matter of great importance to reveal the functional profile of soil microbial communities. The FAPROTAX predicted functional analysis of bacterial communities exhibited the greatest number of OTUs for metabolic processes related to C-cycle and N-cycle and energy production in all soil depth (Fig. 8, Supplementary Fig. 2). Hence, these fundamental resource pathways were considered as potential drivers of bacterial community structure and the key functions of the bacterial community in the red soil [61]. The C- and N-cycles are a matter of great concern to understand their regulation in soil microbial ecology because soil bacterial communities play a key role in the regulation of biogeochemical cycles. Our findings suggest that the functional profile of the bacterial community was significantly shaped by the soil pH [62]. In general, the FAPROTAX analysis revealed the microbial community's expected functions, provided a brief outline of functional potential within the microbial community. Therefore, we recommend that further in-depth studies of metagenomic sequencing are needed for this system to fully evaluate the potential functional gene categories for a comprehensive understanding of the microbial ecology under different soil profiles.

## Conclusions

The ultimate objective of exploring the bacterial diversity is to obtain a deeper understanding of who is there and doing what? To elucidate these questions, reproducible, reliable, quantitative, and statistically effective experimental information is required. Here, 16S rRNA gene high-throughput sequencing technology was deployed to analyze the bacterial diversity in the red soil of the pomelo orchard of Fujian Province. Our results showed that soil depth strongly influenced the soil bacterial communities. The relative abundance of *Proteobacteria*, *Acidobacteria,* and *Bacteriodetes* decreased with increasing soil depth. Principal coordinates analysis also showed that bacterial community composition was significantly affected across the different soil depths. Among the measured edaphic factors, soil pH was the most significant dominant factor in elucidating the dissimilarity in bacterial community composition in red soil. The FAPROTAX revealed that predicted functions of bacterial communities related to the C and N cycle were dominated in the red soil and also significantly affected by the soil pH. Our results indicated that soil pH rather than soil nutrients was the main factor for soil bacterial diversity, community composition, and functional profile in the red soil of pomelo orchard. We recommend that further studies of metagenomics sequencing are needed to evaluate the potential functional gene categories. Overall, these results provide valuable findings regarding the structure and functions of bacterial communities and suggest alleviation of soil acidification by adopting integrated management practices to preserve the soil microbial communities for better ecological functioning.

## Methods

### Sampling site and soil sampling collection

Soil samples were collected from the red soil of pomelo orchards of Pinghe County Fujian province, Southeast China (24°02′–24°35′ N, 116°54′–117°31′ E). This region is characterized by subtropical monsoon climatic conditions with an annual precipitation 1600–2000 mm, and an average annual temperature ranges from 17.5–21.34ºC. The soil type is classified as haplic ferrasol with sand, silt and clay about 39.2%, 35.8%, and 25.1%, respectively [63, 64]. In June 2019, we collected 57 soil samples of the red soil from three different vertical soil depths, i.e., 0–20 cm (RS1), 20–40 cm (RS2), and 40–60 cm (RS3) and each depth contained 22, 17, and 18 soil samples, respectively, with the removal of top 5 cm soil layer to evade the exogenous disturbance. Each soil sample was a composite of 10 subsamples collected from the dripline, and two subsamples from each tree and trees were apart from 3 m from each other. The soil samples were immediately moved to the laboratory on ice. Subsequently, sieved through a mesh of 2 mm diameter, and remaining straw residues and fine roots were removed manually. Each soil sample was separated into two portions, one of which was used to determine soil physicochemical characteristics (stored at 4ºC), and the other part for molecular analyses (stored at -80ºC until use). The detail of intensive input of N.P.K has been shown in Supplementary Table S1.

### Soil physicochemical properties

For determination of soil pH, soil/water (1:2.5, *w/v*) suspension was prepared, and pH meter (ORION A215 STAR, Thermo Ltd., USA) was used for its determination. Available phosphorous (AP) was measured by using sodium bicarbonate (NaHCO_3_, 0.5 mol· L^−1^) at pH 8.5 (2.5 g of soil was mixed with 50 mL solution, and shaken for 30 min) [65]. For Available potassium (AK), ammonium acetate (NH_4_CH_3_CO_2_) extraction and subsequent flame photometer analysis were performed [66]. Available nitrogen (AN) was extracted by sodium hydroxide (NaOH) hydrolysis [67].

### Soil DNA extraction

Total soil DNA was extracted from the 0.5 g of a soil sample by Soil DNA-Extraction-Kit (MO-BIO Laboratories, Carlsbad, CA-USA) using the manufacturer's protocol. The agarose gel-electrophoresis and Nanodrop (Thermo Scientific NanoDrop 2000) were used to detect DNA purity and concentration. An appropriate amount of sample was taken in a centrifuge tube, diluted the sample with sterile water to 10 ng/µl, and stored in a refrigerator (-40ºC) for subsequent analyses.

### PCR assays and high-throughput sequencing

The extracted total soil DNA was further amplified by using the specific primers. The bacterial 16S V4-V5 region primers 515-F (5'-GTGCCAGCMGCCGCGGTAA-3') with 909-R (5'-CCCCGYCAATTCMTTTRAGT-3') were used [68]. The mixture of PCR (25 µl) contained the 1 × -PCR buffer, MgCl_2_ (1.5 mM), deoxynucleoside triphosphate (0.4 μM), TaKaRa Ex-Taq of 0.5 U, 1.0 μM concentration for each primer, and 10 ng of soil genomic DNA. The PCR reaction included the following steps; initial denaturation at 94 °C for 3 min, followed by 30-cycles at 94ºC for 40 s, 56ºC for 60 s, and 72ºC for 10 mint for the final extension. The two PCR reactions were run for each sample and then combine after PCR-amplification. The PCR products were mixed and subjected to gel-electrophoresis (1% agarose); targeted DNA bands were excised and a gel extraction kit was used for purification. For quantification of PCR products, a NanoDrop was used and then pooled together with an equal molar amount from each sample. For sequencing, the samples were prepared according to manufacturer instruction of the TruSeq DNA kit. The constructed library was quantified by Qubit and qPCR and then sent for sequencing on Illumina Miseq system (Bobett Biotechnology Co., Ltd, Sichuan, China).

### Processing of sequencing data

Quantitative Insights Into Microbial Ecology (QIIME v1.9.0) program was used to process the raw sequences with the default settings [69] and UPARSE pipeline [70]. The primers, barcode sequences, and low-quality reads were removed from the analysis. The Operational Taxonomic Units (OTUs) were clustered at 97% sequence similarity. The OTU representative sequence (the sequence with the highest frequency appears in the OTU) was selected and Greengenes was used as reference database for annotation. The sequencing data is available at NCBI BioProject SRA database under the accession number PRJNA714448.

### Data analyses

For downstream statistical analyses, we used the R V_4.0.3 [71]. In total, 4 83 879 high-quality sequences were achieved from all 57 samples with 7914 to 8709 sequences per sample. To strengthen the influence of the sequencing complexity on soil microbial diversity and community composition, the OTUs table was rarified so that each sample having 7900 reads. The Observed, Shannon, Chao1, and ACE indices were used for the determination of species richness and alpha diversity. The Kruskal–Wallis (KW) rank-sum test was used to see if there were any significant differences in alpha diversity between different groups of soil depths. For microbial beta diversity, the principal coordinate analysis (PCoA) based on Bray–Curtis dissimilarity was performed to check the differences in microbial diversity among different soil depths. Furthermore, permutational analysis of variance (PERMANOVA) was performed to assess the significant differences of bacterial communities among different soil depths. The relationship between soil bacterial communities and soil physicochemical parameters, including soil pH, available nitrogen (AN), available phosphorous (AP), and available potassium (AK), was investigated using distance-based redundancy analysis (dbRDA). The mantel test, based on Pearson correlation between soil attributes and Bray–Curtis dissimilarity score, was used to determine which soil factors had a substantial impact on bacterial communities. Correlation analysis between soil properties and the bacterial population is very important in analyzing and inferring the most important factors shaping the community structure. All these statistical analyses were performed using the R package "microeco v0.2.0" [72]. Analysis of variance was applied using Statistix 8 (Version 8.1) to study soil physiochemical properties under different soil depth and significant differences between different soil depths were compared based on least significant difference (LSD, *P* ≤ 0.05).

The co-occurrence network of bacterial communities was determined according to Spearman's correlation. OTUs with significant correlations (*P* < 0.01, ρ˃0.7) were chosen. These correlations were revealed by pairwise analysis of taxa abundance and resulted in a highly complex network thereby each node depicted the phylum, whereas internode (stand among the nodes) represented the significant correlation between the nodes. For visualization and modularity of co-occurrence, Gephi V_0.9.2_ was used. Nodes with a high degree and relative abundance were categorized as keystone species in the co-occurrence network [73]. Finally, for predicting potential functions of soil bacterial community, functional annotation of Prokaryotic taxa (FAPROTAX) was used with default settings [74].

## Supplementary Information

**Additional file 1: Supplementary Figure S1.** The relative abundance of the bacterial communities at the phylum level in different soil profiles. The least significant test (LSD test,* P*<0.05) was applied to check the significance of bacterial relative abundance between the different groups.** Supplementary Figure S2. **Functional analysis of bacterial communities on the basis of % OTUs. The symbol M represents the module, and contains a set of OTUs.** Supplementary Table S1.** The intensive application of N. P. K in Pinghe County.

## Data Availability

All data generated or analyzed during this study are included in this article and its supplementary information files. The raw reads of sequencing data is available at NCBI BioProject SRA database under the accession number PRJNA714448.

## Abbreviations

AN: Available nitrogen
AP: Available phosphorous
AK: Available potassium
RS: Red soil
OTU: Operational taxonomic unit
RA: Relative abundance
PCoA: Principal coordinates analysis
PERMANOVA: Permutational multivariate analysis of variance
dbRDA: Distance-based redundancy analysis
FAPROTAX: Functional annotation of prokaryotic taxa

## References

[1] Zhang M, Muhammad R, Zhang L, Xia H, Cong M, Jiang C (2019). Investigating the effect of biochar and fertilizer on the composition and function of bacteria in red soil. Appl Soil Ecol.

[2] Bardgett RD, Van Der Putten WH (2014). Belowground biodiversity and ecosystem functioning. Nature.

[3] Young IM, Crawford JW (2004). Interactions and self-organization in the soil-microbe complex. Science.

[4] Dhakar K, Pandey A (2020). Microbial Ecology from the Himalayan Cryosphere Perspective. Microorganisms.

[5] Muneer MA, Wang P, Zaib-un-Nisa, Lin C, Ji B (2020). Potential role of common mycorrhizal networks in improving plant growth and soil physicochemical properties under varying nitrogen levels in a grassland ecosystem. Glob Ecol Conserv.

[6] Muneer MA, Tarin MWK, Chen X, Afridi MS, Iqbal A, Munir MZ (2022). Differential response of mycorrhizal fungi linked with two dominant plant species of temperate grassland under varying levels of N-addition. Appl Soil Ecol.

[7] Hao J, Chai YN, Ordóñez RA, Wright EE, Archontoulis S, Schachtman D (2020). The effects of soil depth on the structure of microbial communities in agricultural soils in Iowa.

[8] Seuradge BJ, Oelbermann M, Neufeld JD (2017). Depth-dependent influence of different land-use systems on bacterial biogeography. FEMS Microbiol Ecol.

[9] Maeght J-L, Rewald B, Pierret A (2013). How to study deep roots—and why it matters. Front Plant Sci.

[10] Gu Y, Wang Y, Lu S, Xiang Q, Yu X, Zhao KE (2017). Long-term fertilization structures bacterial and archaeal communities along soil depth gradient in a paddy soil. Front Microbiol.

[11] Blume E, Bischoff M, Reichert JM, Moorman T, Konopka A, Turco RF (2002). Surface and subsurface microbial biomass, community structure and metabolic activity as a function of soil depth and season. Appl Soil Ecol.

[12] Buss HL, Bruns MA, Schultz MJ, Moore J, Mathur CF, Brantley SL (2005). The coupling of biological iron cycling and mineral weathering during saprolite formation, Luquillo Mountains. Puerto Rico Geobiology.

[13] Waring BG, Adams R, Branco S, Powers JS (2016). Scale-dependent variation in nitrogen cycling and soil fungal communities along gradients of forest composition and age in regenerating tropical dry forests. New Phytol.

[14] Wang Y, Zhang H (2016). Physicochemical properties of a red soil affected by the long-term application of organic and inorganic fertilizers. Org Fertil.

[15] Foy CD, Duke JA, Devine TE (1992). Tolerance of soybean germplasm to an acid Tatum subsoil. J Plant Nutr.

[16] Li Y, Han M-Q, Lin F, Ten Y, Lin J, Zhu D-H (2015). Soil chemical properties’, Guanximiyou’pummelo leaf mineral nutrient status and fruit quality in the southern region of Fujian province. China J Soil Sci Plant Nutr.

[17] Zhang S, Yang W, Muneer MA, Ji Z, Tong L, Zhang X (2021). Integrated use of lime with Mg fertilizer significantly improves the pomelo yield, quality, economic returns and soil physicochemical properties under acidic soil of southern China. Sci Hortic (Amsterdam).

[18] Guo JH, Liu XJ, Zhang Y, Shen JL, Han WX, Zhang WF (2010). Significant acidification in major chinese croplands. Science.

[19] Tang W, Shan B, Zhang H, Mao Z (2010). Heavy metal sources and associated risk in response to agricultural intensification in the estuarine sediments of Chaohu Lake Valley. East China J Hazard Mater.

[20] Tarin MWK, Khaliq MA, Fan L, Xie D, Tayyab M, Chen L (2021). Divergent consequences of different biochar amendments on carbon dioxide (CO2) and nitrous oxide (N2O) emissions from the red soil. Sci Total Environ.

[21] Ju X-T, Xing G-X, Chen X-P, Zhang S-L, Zhang L-J, Liu X-J (2009). Reducing environmental risk by improving N management in intensive Chinese agricultural systems. Proc Natl Acad Sci.

[22] Yu C, Hu XM, Deng W, Li Y, Xiong C, Ye CH (2015). Changes in soil microbial community structure and functional diversity in the rhizosphere surrounding mulberry subjected to long-term fertilization. Appl Soil Ecol.

[23] Chen QL, Ding J, Zhu Y-G, He J-Z, Hu H-W (2020). Soil bacterial taxonomic diversity is critical to maintaining the plant productivity. Environ Int.

[24] Lupwayi NZ, Lafond GP, Ziadi N, Grant CA (2012). Soil microbial response to nitrogen fertilizer and tillage in barley and corn. Soil Tillage Res.

[25] Dennis PG, Miller AJ, Hirsch PR (2010). Are root exudates more important than other sources of rhizodeposits in structuring rhizosphere bacterial communities?. FEMS Microbiol Ecol.

[26] Gu Y, Wang J, Cai W, Li G, Mei Y, Yang S (2021). Different amounts of nitrogen fertilizer applications alter the bacterial diversity and community structure in the Rhizosphere Soil of Sugarcane. Front Microbiol.

[27] Omari RA, Sarkodee-Addo E, Fujii Y, Oikawa Y, Bellingrath-Kimura SD (2017). Impacts of fertilization type on soil microbial biomass and nutrient availability in two agroecological zones of Ghana. Agronomy.

[28] Lovell RD, Jarvis SC, Bardgett RD (1995). Soil microbial biomass and activity in long-term grassland: effects of management changes. Soil Biol Biochem.

[29] Ogilvie LA, Hirsch PR, Johnston AWB (2008). Bacterial diversity of the Broadbalk ‘classical’winter wheat experiment in relation to long-term fertilizer inputs. Microb Ecol.

[30] Enwall K, Philippot L, Hallin S (2005). Activity and composition of the denitrifying bacterial community respond differently to long-term fertilization. Appl Environ Microbiol.

[31] Liu J, Sui Y, Yu Z, Shi Y, Chu H, Jin J (2014). High throughput sequencing analysis of biogeographical distribution of bacterial communities in the black soils of northeast China. Soil Biol Biochem.

[32] Delgado-Baquerizo M, Maestre FT, Reich PB, Jeffries TC, Gaitan JJ, Encinar D (2016). Microbial diversity drives multifunctionality in terrestrial ecosystems. Nat Commun.

[33] Muneer MA, Huang X, Hou W, Zhang Y, Cai Y, Munir MZ (2021). Response of fungal diversity, community composition, and functions to nutrients management in red soil. J Fungi.

[34] Newman MEJ (2003). The structure and function of complex networks. SIAM Rev.

[35] Bahram M, Hildebrand F, Forslund SK, Anderson JL, Soudzilovskaia NA, Bodegom PM (2018). Structure and function of the global topsoil microbiome. Nature.

[36] Constancias F, Terrat S, Saby NPA, Horrigue W, Villerd J, Guillemin J (2015). Mapping and determinism of soil microbial community distribution across an agricultural landscape. Microbiologyopen.

[37] Tripathi BM, Kim M, Kim Y, Byun E, Yang J-W, Ahn J (2018). Variations in bacterial and archaeal communities along depth profiles of Alaskan soil cores. Sci Rep.

[38] Eilers KG, Lauber CL, Knight R, Fierer N (2010). Shifts in bacterial community structure associated with inputs of low molecular weight carbon compounds to soil. Soil Biol Biochem.

[39] Liang B, Ma C, Fan L, Wang Y, Yuan Y (2018). Soil amendment alters soil physicochemical properties and bacterial community structure of a replanted apple orchard. Microbiol Res.

[40] Ling N, Chen D, Guo H, Wei J, Bai Y, Shen Q (2017). Differential responses of soil bacterial communities to long-term N and P inputs in a semi-arid steppe. Geoderma.

[41] Hsiao C-J, Sassenrath GF, Zeglin LH, Hettiarachchi GM, Rice CW (2018). Vertical changes of soil microbial properties in claypan soils. Soil Biol Biochem.

[42] Eilers KG, Debenport S, Anderson S, Fierer N (2012). Digging deeper to find unique microbial communities: the strong effect of depth on the structure of bacterial and archaeal communities in soil. Soil Biol Biochem.

[43] Hansel CM, Fendorf S, Jardine PM, Francis CA (2008). Changes in bacterial and archaeal community structure and functional diversity along a geochemically variable soil profile. Appl Environ Microbiol.

[44] Yun Y, Wang H, Man B, Xiang X, Zhou J, Qiu X (2016). The relationship between pH and bacterial communities in a single karst ecosystem and its implication for soil acidification. Front Microbiol.

[45] Schreiter S, Ding GC, Heuer H, Neumann G, Sandmann M, Grosch R (2014). Effect of the soil type on the microbiome in the rhizosphere of field-grown lettuce. Front Microbiol.

[46] Sun R, Zhang XX, Guo X, Wang D, Chu H (2015). Bacterial diversity in soils subjected to long-term chemical fertilization can be more stably maintained with the addition of livestock manure than wheat straw. Soil Biol Biochem.

[47] van der Bom F, Nunes I, Raymond NS, Hansen V, Bonnichsen L, Magid J (2018). Long-term fertilisation form, level and duration affect the diversity, structure and functioning of soil microbial communities in the field. Soil Biol Biochem.

[48] An J, Liu C, Wang Q, Yao M, Rui J, Zhang S (2019). Soil bacterial community structure in Chinese wetlands. Geoderma.

[49] Charokopos N, Artemiou P, Antonitsis P, Rouska E (2010). Repair of aortic regurgitation caused by spontaneous avulsion of aortic valve commissure in a patient with idiopathic thrombocytopenic purpura. Thorac Cardiovasc Surg.

[50] Fernández-Calviño D, Bååth E (2010). Growth response of the bacterial community to pH in soils differing in pH. FEMS Microbiol Ecol.

[51] Sait M, Davis KER, Janssen PH (2006). Effect of pH on isolation and distribution of members of subdivision 1 of the phylum Acidobacteria occurring in soil. Appl Environ Microbiol.

[52] Yang C, Wang X, Miao F, Li Z, Tang W, Sun J (2020). Assessing the effect of soil salinization on soil microbial respiration and diversities under incubation conditions. Appl Soil Ecol.

[53] Wang Q, Wang C, Yu WW, Turak A, Chen D, Huang Y (2018). Effects of nitrogen and phosphorus inputs on soil bacterial abundance, diversity, and community composition in chinese fir plantations. Front Microbiol.

[54] Guo J, Yang J, Zhang L, Chen H, Jia Y, Wang Z (2019). Lower soil chemical quality of pomelo orchards compared with that of paddy and vegetable fields in acidic red soil hilly regions of southern China. J Soils Sediments.

[55] Gui H, Fan L, Wang D, Yan P, Li X, Zhang L (2021). Organic management practices shape the structure and associations of soil bacterial communities in tea plantations. Appl Soil Ecol.

[56] Faust K, Raes J (2012). Microbial interactions: from networks to models. Nat Rev Microbiol.

[57] Santolini M, Barabási AL (2018). Predicting perturbation patterns from the topology of biological networks. Proc Natl Acad Sci U S A.

[58] Nelson MB, Martiny AC, Martiny JBH (2016). Global biogeography of microbial nitrogen-cycling traits in soil. Proc Natl Acad Sci U S A.

[59] Gibbons SM (2017). Microbial community ecology: Function over phylogeny. Nat Ecol Evol.

[60] Louca S, Jacques SMS, Pires APF, Leal JS, González AL, Doebeli M (2017). Functional structure of the bromeliad tank microbiome is strongly shaped by local geochemical conditions. Environ Microbiol.

[61] Hu Y, Bai C, Cai J, Dai J, Shao K, Tang X (2018). Co-occurrence network reveals the higher fragmentation of the bacterial community in Kaidu River than its tributaries in northwestern China. Microbes Environ.

[62] Wang CY, Zhou X, Guo D, Zhao JH, Yan L, Feng GZ (2019). Soil pH is the primary factor driving the distribution and function of microorganisms in farmland soils in Northeastern China. Ann Microbiol.

[63] Yan X, Yang W, Muneer MA, Zhang S, Wang M, Wu L (2021). Land-use change affects stoichiometric patterns of soil organic carbon, nitrogen, and phosphorus in the red soil of Southeast China. J Soils Sediments.

[64] Huang X, Muneer MA, Li J, Hou W, Ma C, Jiao J (2021). Integrated Nutrient Management Significantly Improves Pomelo (Citrus grandis) Root Growth and Nutrients Uptake under Acidic Soil of Southern China. Agronomy.

[65] Yan X, Yang W, Chen X, Wang M, Wang W, Ye D (2020). Soil Phosphorus Pools, Bioavailability and Environmental Risk in Response to the Phosphorus Supply in the Red Soil of Southern China. Int J Environ Res Public Health.

[66] Tan KH. Soil sampling. Prep Anal 2005.

[67] Cornfield AH (1960). Ammonia released on treating soils with N sodium hydroxide as a possible means of predicting the nitrogen-supplying power of soils. Nature.

[68] Tuan NN, Chang YC, Yu CP, Huang SL (2014). Multiple approaches to characterize the microbial community in a thermophilic anaerobic digester running on swine manure: A case study. Microbiol Res.

[69] Caporaso JG, Kuczynski J, Stombaugh J, Bittinger K, Bushman FD, Costello EK (2010). QIIME allows analysis of high-throughput community sequencing data. Nat Methods.

[70] Edgar RC (2013). UPARSE: Highly accurate OTU sequences from microbial amplicon reads. Nat Methods.

[71] Team RC (2014). A language and environment for statistical computing.

[72] Liu C, Cui Y, Li X, Yao M. microeco: An R package for data mining in microbial community ecology. FEMS Microbiol Ecol. 2021;97(2):fiaa255. 10.1093/femsec/fiaa255. 10.1093/femsec/fiaa255 33332530

[73] Zhang L, Tu D, Li X, Lu W, Li J (2020). Impact of long-term industrial contamination on the bacterial communities in urban river sediments. BMC Microbiol.

[74] Louca S, Parfrey LW, Doebeli M (2016). Decoupling function and taxonomy in the global ocean microbiome. Science.

