# Supplementary material for: Soil pH: a key edaphic factor regulating distribution and functions of bacterial community along vertical soil profiles in red soil of pomelo orchard
Source: BMC Microbiol. 2022 Feb 2;22:38. doi: 10.1186/s12866-022-02452-x (PMC8808772; doi:10.1186/s12866-022-02452-x)
Supplement: Supplementary file 1 — Additional file 1: Supplementary Figure S1. The relative abundance of the bacterial communities at the phylum level in different soil profiles. The least significant test (LSD test, P<0.05) was applied to check the significance of bacterial relative abundance between the different groups. Supplementary Figure S2. Functional analysis of bacterial communities on the basis of % OTUs. The symbol M represents the module, and contains a set of OTUs. Supplementary Table S1. The intensive application of N. P. K in Pinghe County. [file 12866_2022_2452_MOESM1_ESM.docx]

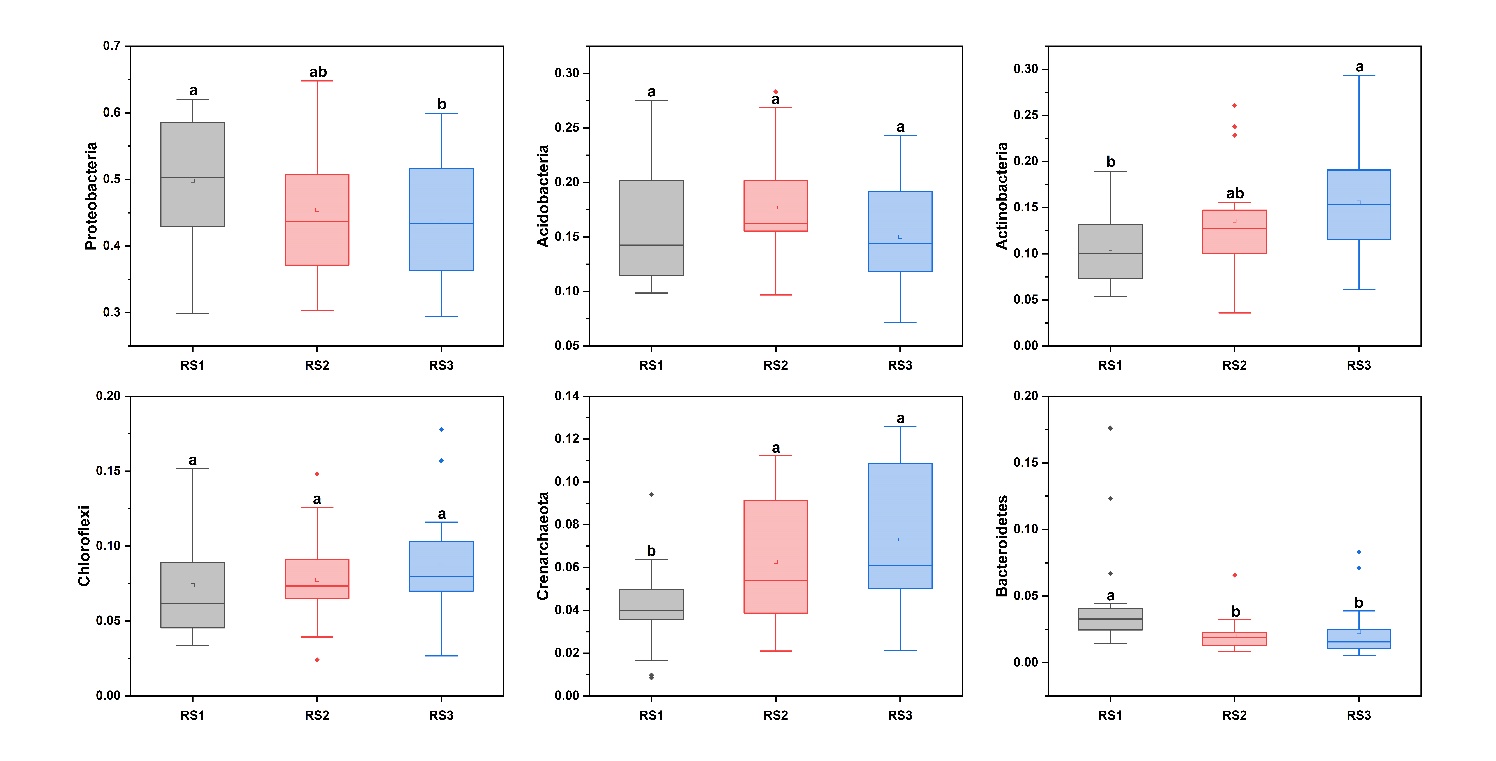


**Supplementary Figure S1.** The relative abundance of the bacterial communities at the phylum level in different soil profiles. The least significant test (LSD test, *P*<0.05) was applied to check the significance of bacterial relative abundance between the different groups.


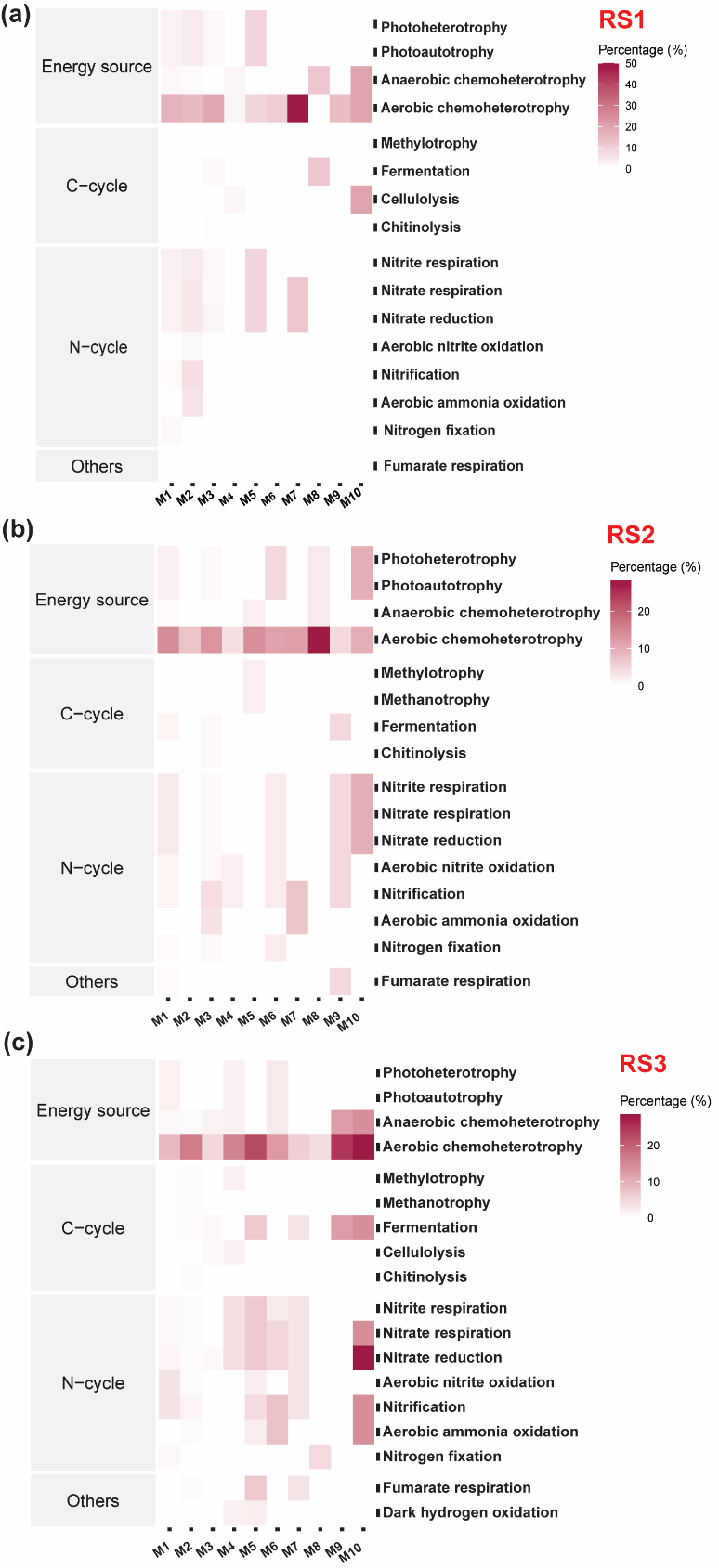


**Supplementary Figure S2.** Functional analysis of bacterial communities on the basis of % OTUs. The symbol M represents the module, and contains a set of OTUs.

**Supplementary Table S1**. The intensive application of N. P. K in Pinghe County.

| \| **Nutrient type** \| **Nutrient input (Kg·ha^-1^)** \| \| --- \| --- \| \| N \| 1206±508 \| \| P2O5 \| 971±431 \| \| K2O \| 955±368 \| |  |
| --- | --- | --- | --- | --- | --- | --- | --- | --- | --- |
